# Periacetabular osteotomy using computed tomography-based navigation: preoperative planning and accuracy evaluation

**DOI:** 10.1007/s11548-024-03210-2

**Published:** 2024-06-18

**Authors:** Yutaka Inaba, Taro Tezuka, Masatoshi Oba, Hyonmin Choe, Hiroyuki Ike

**Affiliations:** Department of Orthopedic Surgery, Yokohama City University, 3-9 Fukuura, Kanazawa-ku, Yokohama, Japan

**Keywords:** Rotational acetabular osteotomy, Developmental dysplasia of the hip, Computed tomography-based navigation

## Abstract

**Purpose:**

Since 2011, we have used computed tomography (CT)-based navigation to perform safe and accurate rotational acetabular osteotomy (RAO) for treating developmental dysplasia of the hip. We developed a new method with four fiducial points to improve the accuracy of a published technique. In this study, we introduced a new method to achieve reorientation in accordance with planning and evaluated its accuracy.

**Methods:**

This study included 40 joints, which underwent RAO used CT-based navigation. In 20 joints, reorientation was confirmed by touching the lateral aspect of the rotated fragment with navigation and checking whether it matched the preoperative plan. A new fiducial point method was adopted for the remaining 20 joints. To assess the accuracy of the position of the rotated fragment in each group, postoperative radial reformatted CT images were obtained around the acetabulum and three-dimensional evaluation was performed. The accuracy of acetabular fragment repositioning was evaluated using the acetabular sector angle (ASA).

**Results:**

The absolute value of ΔASA, which represents the error between preoperative planning and the actual postoperative position, was significantly smaller in the new fiducial method group than the previous method group in the area from 11:30 to 13:30 (*p* < 0.05). The Harris Hip Score at 1 year after surgery did not differ significantly between the previous and new fiducial point methods.

**Conclusion:**

The new fiducial point method significantly reduced reorientation error in the superior-lateral area of the acetabulum: significantly fewer errors and fewer cases of under-correction of lateral acetabular coverage were recorded. The four-reference fiducial method facilitates reorientation of the acetabulum as planned, with fewer errors. The effect of the improved accuracy of the fiducial point method on clinical outcomes will be investigated in the future work.

## Introduction

Developmental dysplasia of the hip (DDH) causes hip pain and osteoarthritis (OA). DDH is a major cause of hip OA in the Japanese population [1]. Dysplasia of the acetabular roof induces instability and incongruence of the hip joints at an early age, which triggers the progression of hip OA and may require total hip arthroplasty (THA) even in young or middle-aged patients. Since THA in young or middle-aged active patients is associated with high rates of loosening [2, 3], joint-preserving operation is an important surgical option.

Rotational acetabular osteotomy (RAO) is a periacetabular osteotomy [4–8] developed by Ninomiya and Tagawa [9] that corrects insufficient femoral coverage by rotating the acetabular roof with the articular cartilage. Appropriate surgical techniques and patient selection are important to achieve successful outcomes. Under-correction of the acetabular roof may result in hip joint instability; hence, sufficient coverage of the acetabular roof is essential in this procedure. RAO is a technically difficult procedure that involves a large skin incision and intraoperative radiation exposure for fluoroscopy. Since 2011, we have been using computed tomography (CT)-based navigation (OrthoMap 3D^Ⓡ^, Stryker Orthopedics, Mahwah, NJ, USA) for RAO to perform safe and accurate osteotomy [10]. The use of CT-based navigation eliminates intraoperative radiation exposure and enables surgery with a smaller incision. CT-based navigation enables intraoperative tracking of the tip of the chisel and allows for the performance of safe and accurate osteotomy. However, upon moving the acetabular fragment after the completion of osteotomy, the bony fragment itself cannot be tracked in real time with navigation. Previously, reorientation was confirmed by tracing the lateral aspect of the rotated fragment with navigation and checking whether it matched the preoperative plan. However, confirmation after reorientation was not precise enough, which could result in substantial error. Therefore, we developed a new method to achieve more accurate rotation.

Two-dimensional radiological evaluation methods, such as the acetabular head index (AHI) and center edge (CE) angles, are used commonly; however, these measurement methods are reportedly inaccurate for evaluating coverage of the femoral head with the rotated fragment compared with three-dimensional (3D) measurement methods [11]; however, only a few 3D methods are available to evaluate acetabular coverage.

Tanaka et al. compared 3D acetabular bone coverage before and after RAO using their original software [12], and Cheng et al. measured the area of femoral head surface covered by the acetabulum using 3D CT-based measurement [13]. These studies evaluated the area of the femoral head covered by the acetabulum. DeFroda SF. et al. examined the percentage of acetabular coverage of the femoral head using custom software. In this study, the area of coverage and the radial coverage of the acetabulum along the clock face were calculated [14]. In the present study, we developed a method to evaluate the preoperative and actual postoperative acetabular morphology in three dimensions using two types of open-source software (OSS).

The purposes of this study are to introduce a new method to determine reorientation of the rotational bone fragments during the RAO procedure and to report the results of 3D evaluation to determine the accuracy of reorientation of the rotational bone fragments using two different OSSs.

## Patients and methods

The study was approved by the institutional review board, and informed consent was obtained by giving patients the opportunity to opt out of the study on the university’s website. Since we started using CT-based navigation for RAO in 2011, 137 navigated RAOs have been performed. The new method was applied to 20 cases with a minimum follow-up period of 1 year until now; therefore, we extracted 20 cases treated with the previous method as controls using propensity score matching for the preoperative Harris Hip Score (HHS), CE angle, AHI, and acetabular roof obliquity (ARO) to compare the accuracy of the new method with that of the previous method.

### Preoperative planning and procedure of RAO

During preoperative planning, Digital Imaging and Communication in Medicine (DICOM) data of the hip CT scans were converted into a 3D model using a dedicated software (Zed Hip, LEXI, Japan) to create an osteotomy model. The osteotomy model was converted into a Standard Triangulated Language (STL) file using Geomagic Freeform (3D System Corporation, Rock Hill, SC, USA) and was imported into OrthoMap. For preoperative planning, a spherical osteotomy line was envisaged for the acetabulum such that the osteotomy line passed approximately 25 mm proximal to the upper acetabular margin, from the innominate groove of the ischium to the midpoint between the greater sciatic notch and posterior acetabular margin, with the center of the sphere located near the center of the femoral head (or center of the hip joint). The line was planned such that it ran distal to the anterior inferior iliac spine to the center of the pubis and slightly through the iliac inner cortex to avoid cutting into the joint. The diameter of the sphere was generally 80–90 mm and differed among cases.

The amount of rotation of the acetabular fragment after osteotomy was planned until the ARO was 0° in the mid-frontal plane, and the anterior CE angle was 45–50 degrees. The position of the femoral head was planned so that the distance between the center of the femoral head and the Koehler line was 35–40 mm. These values were slightly modified according to the morphology of the patient.

During surgery, the osteotomy line was marked on the lateral side of the ilium using navigation imaging guidance in line with the preoperative plan. The tip of the curved chisel was then registered, and pelvic osteotomy was performed while checking the position of curved chisel tip in real time. After osteotomy, the free fragment was rotated anterolaterally, and absorbable screws were used to fix the rotational fragment.

**Fig. 1:**
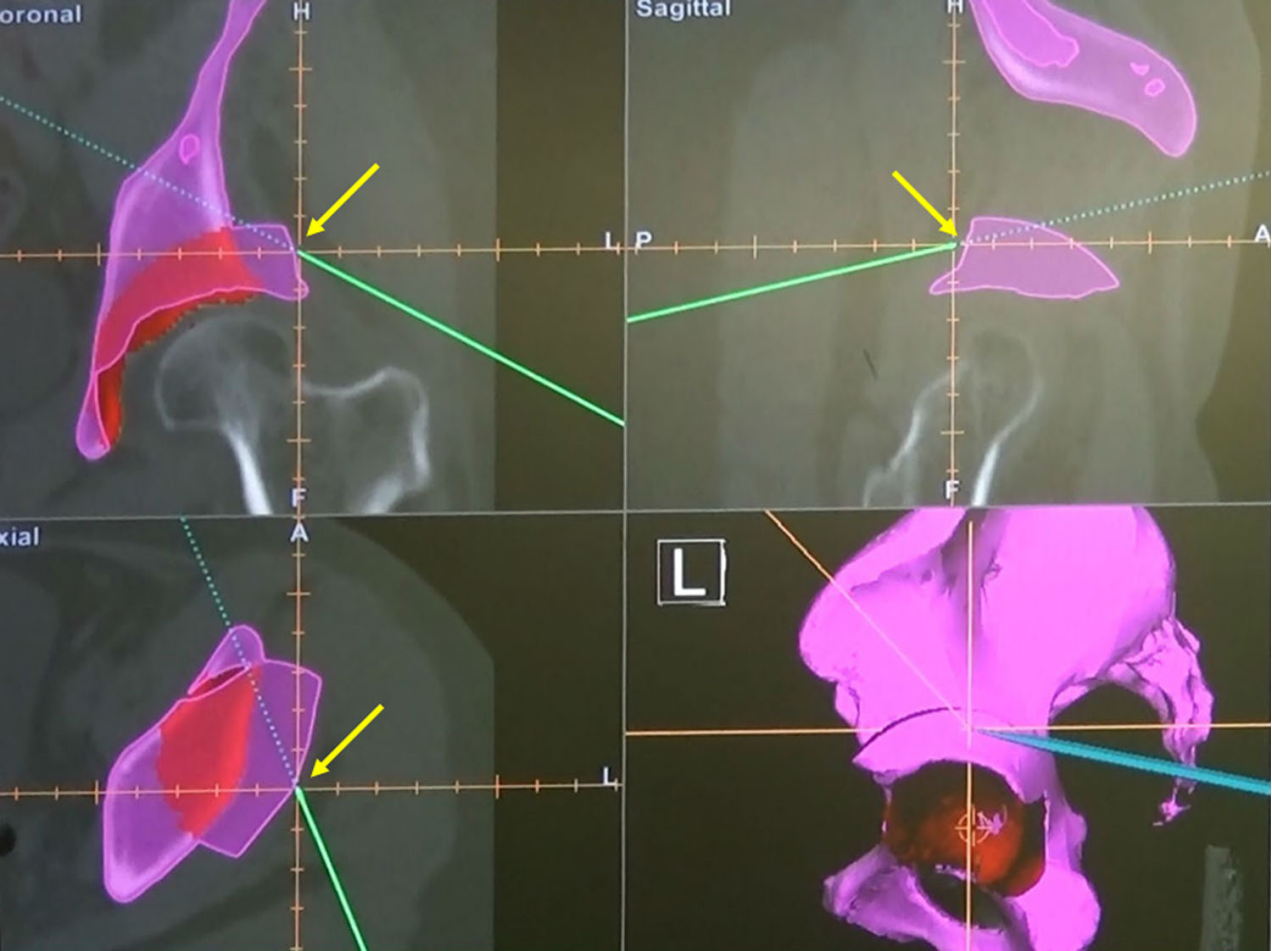
Confirmation of the position of the bone fragment after transposition in the previous method. To confirm whether or not the position fits the surface of the planned position after rotation in the preoperative plan, the surface of the rotated bone fragment was touched with a pointer (green bar) after rotation. The yellow arrows show the pointer tip, which touches the surface of the rotated bone fragment at the same position as in the preoperative plan (purple portion)

Before the introduction of the new method, the lateral aspect of the rotational fragment was verified after rotation of the bony fragment using navigation to confirm that it was in the preoperative planned position, as described in our previous study [10] (Fig. 1).

**Fig. 2:**
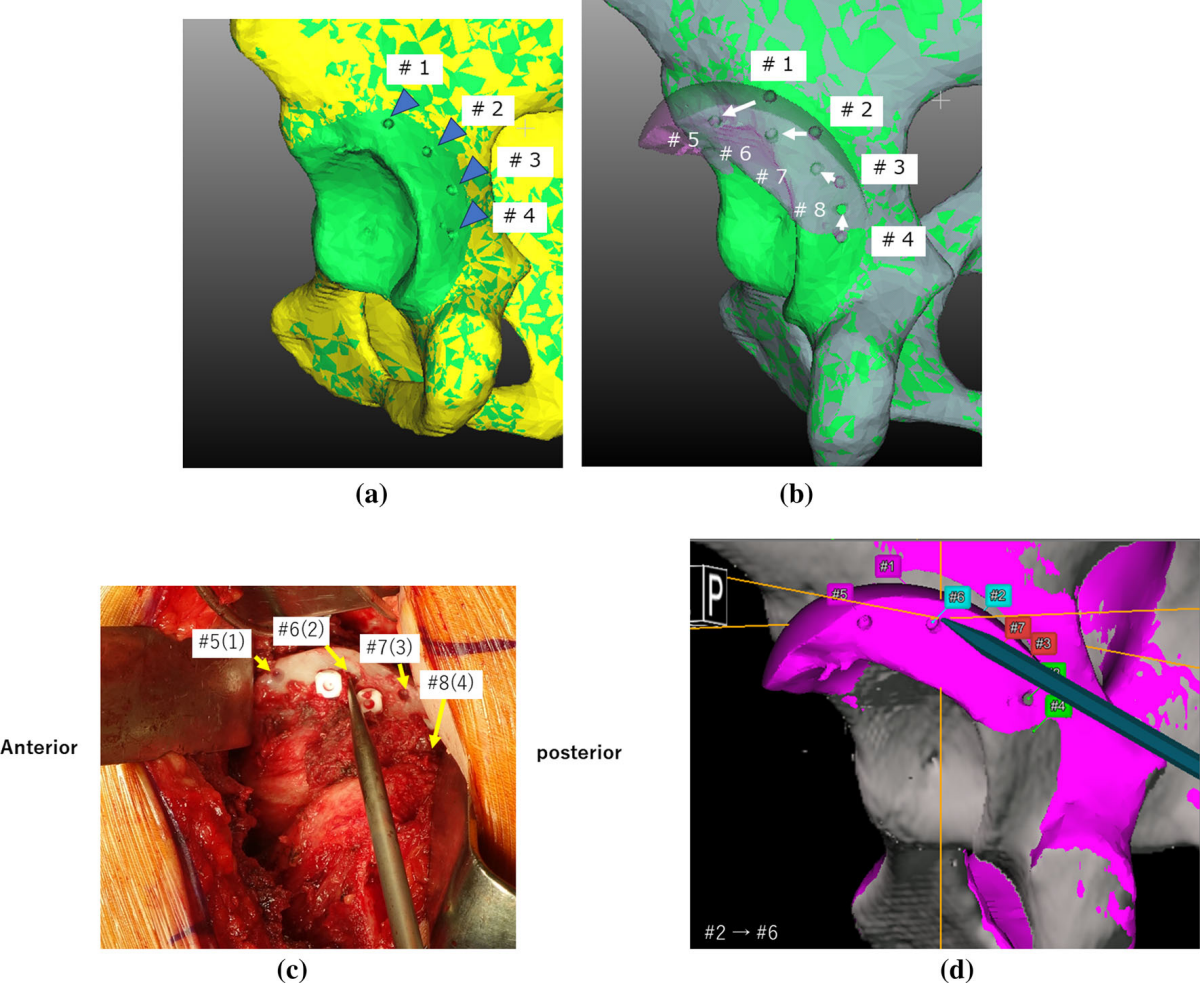
Fiducial point method. **a** Making four fiducials on the planning model. In the preoperative plan, four 2 mm diameter notches (fiducials: #1–4, blue arrows) are made around the acetabulum in the preoperative planning model before virtual osteotomy. **b** Relocation of fiducials following vertical osteotomy. Four fiducials are relocated with the movement of bone fragments in the virtually osteotomized planning model. The relocated position of #1 that moves after rotation is defined as #5. Similarly, #2 is defined as #6, #3 as #7, and #4 as #8 after rotation. **c** Confirmation of position after transposition of the bone fragment with fiducials. Intraoperatively, the fragment position is confirmed using four fiducials and reorientation is performed by confirming that all four fiducials match the relocation corresponding points. Fiducial #2 moves to its corresponding point (#6) after transposition and the bone fragment is fixed with absorbable screws. **d** Intraoperative confirmation of the position of the fiducials on a computer screen. This figure shows the computer screen during procedure described in (**c**). On the computer screen, it is confirmed that the tip of the pointer has touched point #6, the point corresponding to #2

In the new method, an additional step was implemented to create four 2 mm diameter notches (fiducials) around the acetabulum in the preoperative 3D model (Fig. 2a). Thereafter, virtual osteotomy was performed to create a planning model that contained the above-mentioned fiducials. Four fiducials were relocated with the movement of the bony fragments after virtual osteotomy. The relocated position of fiducial #1 was defined as #5, and similarly, fiducial #2 was defined as #6, fiducial #3 as #7, and fiducial #4 as #8 (Fig. 2b). Intraoperatively, these four fiducials were created around the acetabular rim using a high-speed bur under navigation assistance before osteotomy. The fragment position was confirmed using these fiducials, and reorientation was performed by confirming that all four fiducials matched the corresponding relocation points (Fig. 2c, d).

The 20 joints in which reorientation was confirmed by tracing the lateral aspect of the rotated fragment with navigation were designated as the “previous method” group, and the 20 hips which were subjected to the new method were designated as the fiducial point method group.

Table 1 shows the patients’ demographic data and preoperative radiographic measurements. No significant differences were found between the two groups due to propensity matching. The primary endpoint of this study was to evaluate whether there was a difference in the preoperative planned and postoperative measured angles between the two groups. The secondary radiological endpoints were the postoperative CE angle, AHI, and ARO, and the clinical endpoint was the HHS at 1 year postoperatively.

**Table 1:** Patients’ demographic data and preoperative radiographic measurements

	Previous group	Fiducial point method group	*p*
n	20	20	N.A
Age (years)	39 ± 12	38 ± 11	N.S
BMI (kg/m^2^)	22 ± 8	23 ± 7	N.S
Preoperative CE angle (degrees)	11 ± 5	10 ± 6	N.S
Preoperative AHI (%)	62 ± 7	58 ± 8	N.S
Preoperative ARO (degrees)	26 ± 7	25 ± 8	N.S

*AHI* acetabular head index, *ARO* acetabular obliquity angle, *BMI* body mass index, *CE angle* center edge angle, *N.A*. not available, *N.S*. not significant

### 3D evaluation of reorientation of bony fragments

CT images of the hip joints acquired preoperatively and at 1 week postoperatively were used for image analysis. A Sensation 16® (Siemens, Munich, Bavaria Germany) CT scanner was used, and the slice thickness was 1.5 mm. Preoperative CT images of the pelvis were loaded onto 3D Slicer (www.slicer.org/) [15], and a 3D model was created by segmenting the pelvis and proximal femur (preoperative model). Based on the preoperative model, virtual RAO was performed on a computer and a model was created for intraoperative navigation (planning model). This planning model was used to perform the RAO. CT was performed again at 1 week postoperatively, and a 3D model of the pelvis and proximal femur was created from the CT images using the same procedure as that before surgery (postoperative model).

**Fig. 3:**
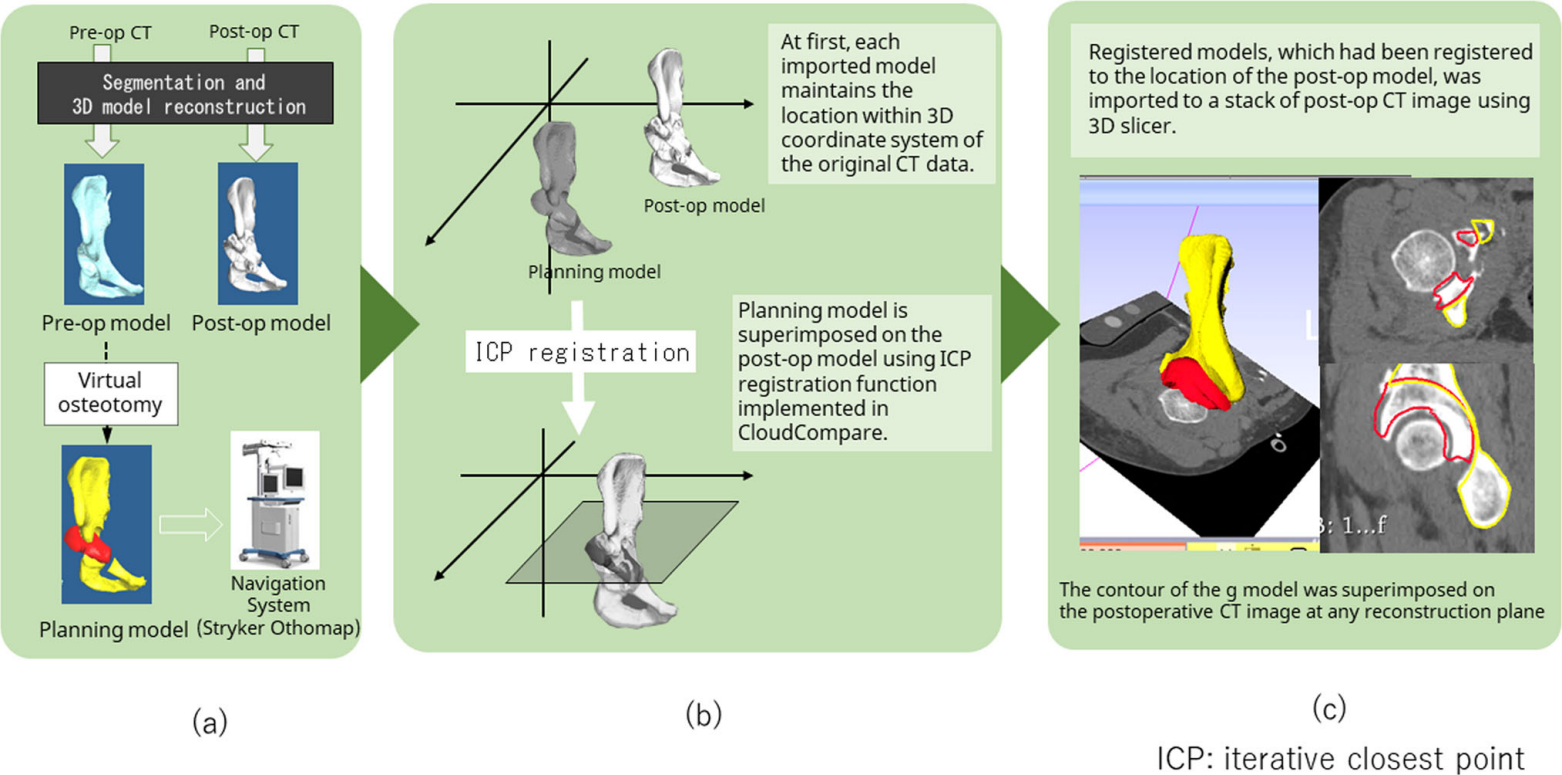
Use of the model-based registration method to compare the preoperative planned and postoperative models. **a** A three-dimensional (3D) model of the pelvis and proximal femur is created based on the computed tomography (CT) images obtained before and after surgery. **b** First, each imported model maintains the location within the 3D coordinate system of the original CT data. The antero-pelvic plane was represented as an *X*–*Y* plane, and the line passing through the bilateral center of the femoral heads was denoted as the *X*-axis, while the axis vertical to *X*–*Y* plane passing through the femoral head of operative side was denoted as the *Z*-axis. The planned model is superimposed over the postoperative model using the iterative closest point (ICP) registration function implemented in CloudCompare. **c** Registered models, which had been registered to the location of the postoperative model, were imported to a stack of postoperative CT images using 3D Slicer; this software enables the display of the cross section of the pelvis in any plane

The preoperative planned and postoperative measured angles were evaluated using the following two OSSs: a 3D slicer was used to reconstruct the pelvis model from the CT image data, to superimpose the DICOM data and 3D models, and to reconstruct radial section images from normal axial images. CloudCompare 2.6.3 software (www.cloudcompare.org) [16] was then used to calculate the femoral head center position or to register the pre- and post-3D model of the pelvis. The flowchart of image analysis is shown in Fig. 3. First, a 3D model of the pelvis and proximal femur was created based on CT images obtained before and after surgery (Fig. 3a). Next, the preoperative and postoperative image data were registered. The coordinate system used for the measurement was set up using CloudCompare (Fig. 3b). Finally, the registered model was superimposed onto the CT images to perform the measurements (Fig. 3c).

The planning and postoperative models were loaded into CloudCompare and displayed at different positions in the 3D space, reflecting the position of the pelvis at the time of the CT scan. To accurately superimpose the planning and postoperative models, registration using the iterative closest point algorithm for CloudCompare was used. The planning and postoperative models were moved, and when the mean-squared error of the points that made up each model was 1.0E−5 or less, the planning and postoperative models were considered to exhibit complete superimposition.

After the 3D models were superimposed, the planning model was saved in STL format and imported into 3D Slicer simultaneously with the postoperative CT image. The planning model was displayed at the position of the pelvis on the postoperative CT image. The contours of the model were displayed in all arbitrary cross sections of the postoperative CT image reconstructed by the 3D slicer, and this was used to measure and compare the planning model and postoperative acetabular morphology.

**Fig. 4:**
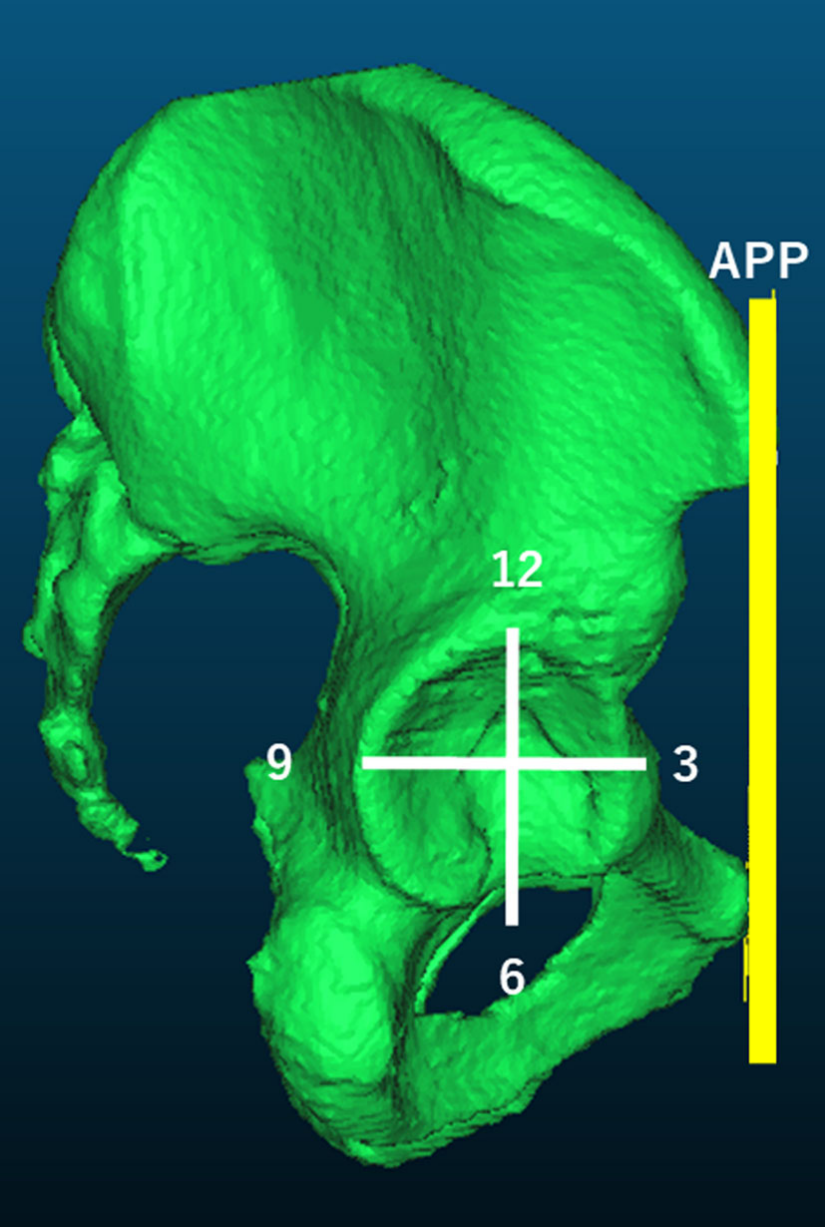
Coordinate system of the acetabulum for 3D measurements. For setting the coordinate system, a line passing through the center of the femoral head and parallel to the anterior pelvic plane is defined as 12:00, and anterior was defined as 3 and posterior as 09:00

**Fig. 5:**
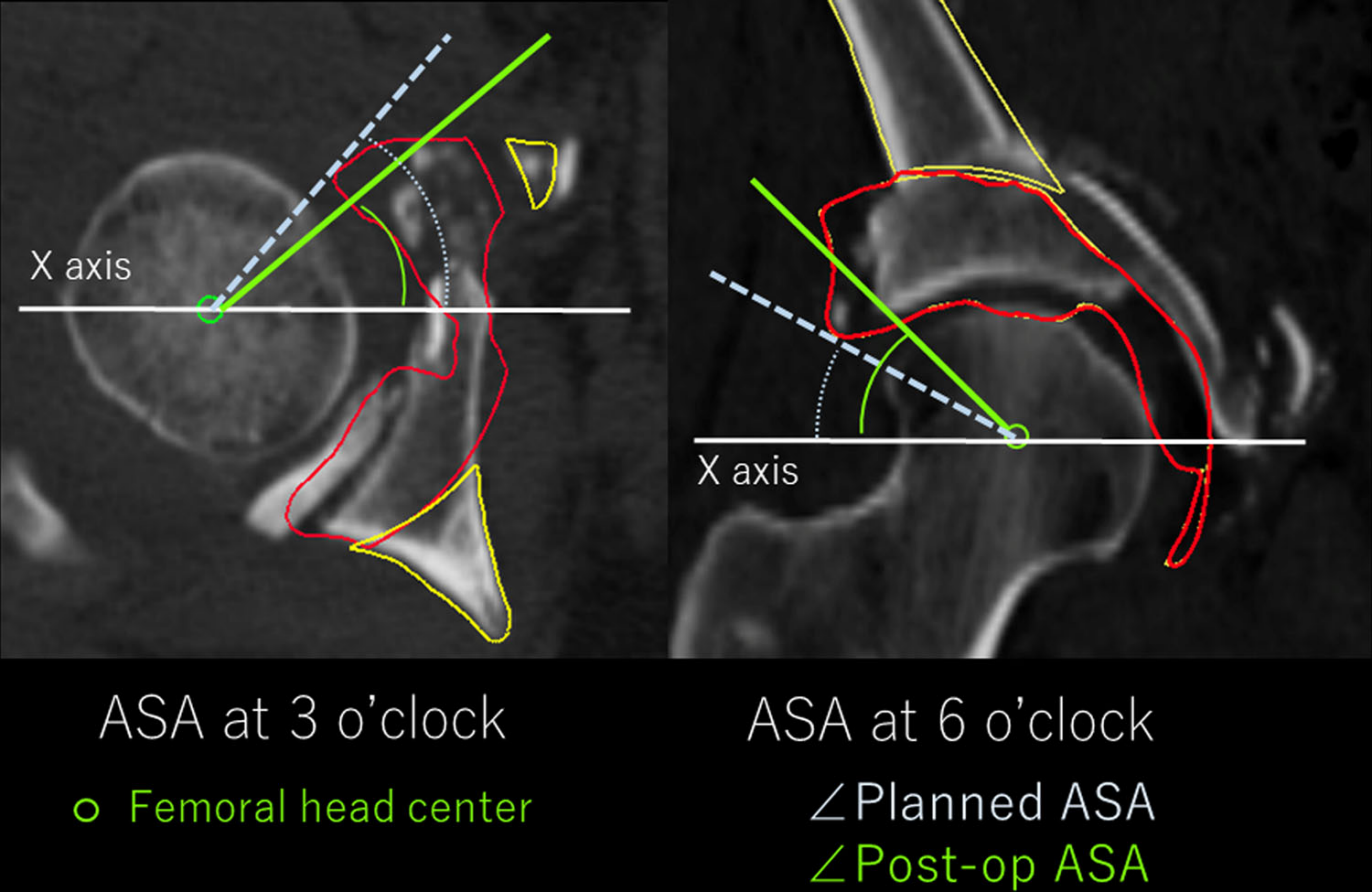
Definition of acetabular sector angle (ASA): index of acetabular coverage. The ASA was defined as the angle between the line drawn from the acetabular rim to the femoral head center and the *X*-axis on every radial reconstruction image. Figure shows the ASA at the 3:00 position using postoperative CT superimposed with the planning model. The red line indicates the planning model. The angle made by the *X*-axis and the line connecting the femoral head center and acetabular rim in planning (dotted line) is the planned ASA. The angle made by the X-axis and the line connecting the femoral head center and actual post-operative acetabular rim (green line) is the postoperative ASA. **a** Shows the ASA at 3 o’clock and **b** shows the ASA at 12 o’clock. The difference between the planned and postoperative ASAs is denoted as ΔASA, i.e., the index of accuracy in acetabular fragment reorientation. If the ΔASA is positive, it means that the postoperative acetabular coverage exceeds the preoperative plan. Conversely, if the ΔASA is negative, it implies under-correction compared to the preoperative plan

A 3D Slicer function was used to establish the coordinate system with the anterior pelvic plane (APP) as a reference. The center of the femoral head, which was used as the reference point for the measurement (Fig. 4), was determined by approximating the femoral head to a best-fit sphere. To evaluate 3D femoral head coverage, the planning model after superimposition was imported into the postoperative pelvic image on 3D Slicer, and a clock face system was established to measure femoral head coverage with the APP plane at the 12 o'clock cephalad (*Z*-axis direction), 3 o'clock anterior, and 9 o’clock posterior positions. Radial reconstruction images were obtained at every 15° (every 30 min) around the *X*-axis passing through the center of the femoral head. The acetabular sector angle (ASA), an index of 3D femoral head coverage, was defined as the angle between the line drawn from the acetabular rim to the femoral head center and the *X*-axis in every 30 min radial reconstruction image (Fig. 5).

The ASA was measured in the same manner as that in the planning model. The coordinates of the femoral head center of the preoperative model were determined by calculating the optimal sphere of the head, as in the postoperative model. The femoral head center of the planning model was determined with reference to the joint gap between the preoperative femoral head and acetabulum, and the ASA of the planning model was measured based on this gap. At each measurement point (every 30 min), the planning and postoperative ASAs were compared using Student’s unpaired t test. In addition, the difference between the ASA value obtained postoperatively and during planning was calculated and defined as ΔASA (postoperative ASA–planning model ASA). If the ΔASA was positive, it meant that the postoperative acetabular coverage was excessive compared to the preoperative plan. Conversely, if the ΔASA was negative, it implied under-correction compared to the preoperative plan. For evaluation of the accuracy of the conventional and fiducial point groups, the ΔASA in each group was compared using Student’s unpaired t test. A *p*-value of less than 0.05 was considered significant. The power analysis showed a power of 0.78 for the 20 cases in each group with significance level (*α*) of 0.05 and an effect size of 0.8, which is appropriate for analysis.

## Results

**Fig. 6:**
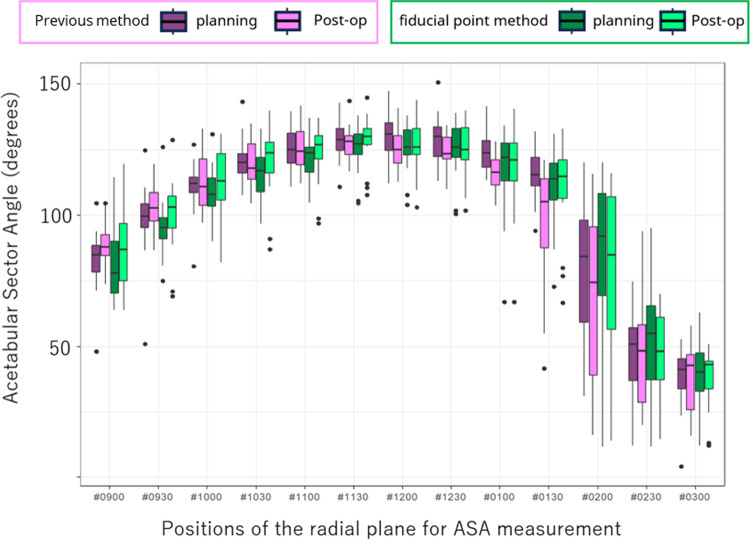
Planned and postoperative (actual) acetabular sector angles (ASAs) at 13 radial reformat planes (9–3 o’clock position). The postoperative ASA values did not differ significantly between the two groups

**Fig. 7:**
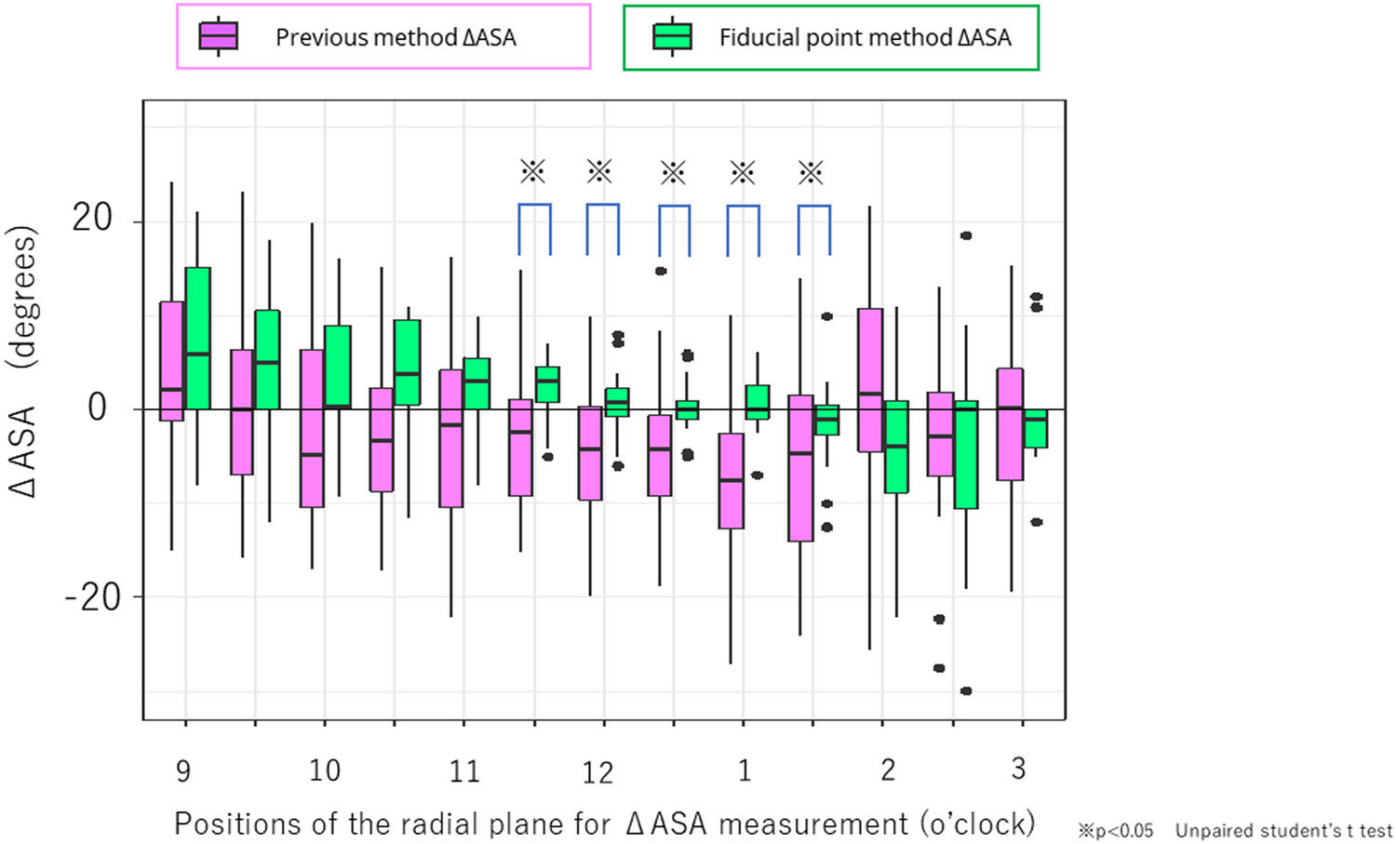
Difference between the planned and postoperative acetabular sector angles. The fiducial point method executed the preoperative plan more accurately in the anterolateral part of the acetabulum (11:30–13:30) compared with the previous method

The preoperative planned and actual postoperative ASA values are presented as a box chart in Fig. 6. The postoperative ASA values did not differ significantly between the two groups. Figure 7 shows the ΔASA of the two groups from 9:00 to 3:00. The absolute value of ΔASA, which represents the error between preoperative planning and the actual postoperative position, was significantly smaller in the fiducial method group than the previous method group in the area from 11:30 to 13:30 (p < 0.05). The fiducial point method executed the preoperative plan more accurately in the anterolateral part of the acetabulum than the previous method.

Table 2 shows the HHS at 1 year after surgery and the postoperative radiographic angles of the two groups. Bony coverage improved significantly after surgery in each group, and the HHS after surgery was also satisfactory in both groups. There were no significant differences in the radiographic measurements and clinical scores between the two groups.

**Table 2:** HHS at 1 year after surgery and postoperative radiographic measurements

	Previous group	Fiducial point method group	*p*
HHS (points)	91 ± 9	90 ± 10	N.S
CE angle (degrees)	33 ± 7	32 ± 5	N.S
AHI (%)	88 ± 7	89 ± 8	N.S
ARO (degrees)	8.5 ± 5	6.2 ± 7	N.S

*AHI* acetabular head index, *ARO* acetabular obliquity angle, *CE angle* center edge angle, *HHS* Harris hip score, *N.S*. not significant

## Discussion

In navigation-assisted RAO performed at our institution, osteotomy is performed along the preoperative planned osteotomy line indicated by imaging guidance [10]; moreover, several studies have reported on the use of computer navigation for periacetabular osteotomy [17–22]. Recently, 3D quantitative assessment methods have been devised [13, 14], and the evaluation of 3D acetabular morphology is becoming increasingly important. This facilitates precise performance of osteotomy; however, the bony fragment itself cannot be tracked in real time with navigation, and accuracy in this case is problematic owing to the movement of the bone fragments. To solve this problem, Pflugi et al. reported the use of an augmented marker attached to both the pelvis and fragment to track the position of the resected rotational fragment [23], while Grupp et al. reported the use of pose estimation [24]. Racdt et al. performed intraoperative acetabular ostectomy with intraoperative acetabular external notches around the acetabulum [25]. They created cortical notches around the acetabulum as reference fiducials intraoperatively and used a biomechanical guidance system to automatically calculate the diagnostic angles.

In the present study, our fiducial point method yielded a significant improvement in loading surface coverage compared with that of the previous method from 11:30 to 13:30. This may be attributed to the fact that previous methods allowed us to confirm the location of the lateral aspect of the bony fragment but did not permit determination of the amount of movement of a specific point on a bony fragment. In contrast, the fiducial points methods allowed us to check whether a specific point in the preoperative plan is actually reached. In the current study, although the four fiducial points were located close to each other, ideally, they should be set apart around the acetabulum. This was difficult because we adopted minimally invasive surgery with a skin incision of less than 10 cm and the placement of fiducial points was limited to the superior lateral aspect of the acetabulum, considering the thickness of rotational bony fragment. However, the new method introduced in this study with four reference fiducials is simple and does not require the installation of an antenna on the rotated fragment or biomechanical guidance if CT-based navigation is available, and modification of the navigation system is not necessary. One limitation is that real-time tracking of bone fragment movement is not possible with this method, and movement of the bone fragment is performed manually. Inexperienced surgeons may not be able to intuitively determine the amount of bone fragment movement and may take time to determine the position of the bone fragment. However, there is no commercially available system that can confirm the position of bone fragments in real time. We believe that this principle is effective because it allows easy intraoperative confirmation of the position of the bone fragments in three dimensions. We also believe that this system will be an effective educational tool for inexperienced surgeons.

For 3D evaluation, this study used two OSSs, viz. 3D Slicer and CloudCompare, for image measurement and analysis. The method presented in this paper enables the evaluation of preoperative and postoperative bone morphologies in three dimensions, the most important feature of which is the use of an OSS. For 3D image analysis similar to that used in this study, the use of image analysis software is usually onerous. However, many facilities either do not own such software or have limited terminals on which it can be installed, limiting its use. The two OSSs used in this study can be downloaded free of charge; therefore, any facility with computers with a certain level of performance can perform the same measurements and analyses, akin to this study. The volume registration method used in this study can be applied to bones other than the pelvis and femur to compare bone morphology at different time points in the same patient. The advantage of OSS over commercial software is that it can be used free of charge, and its functionality can be extended by installing plug-ins developed by volunteers. In terms of the measurement method, DeFroda et al. examined 3D acetabular coverage in 30 patients with femoroacetabular impingement, and acetabular coverage was measured using the points on the acetabular rim along planes around the normal line of the acetabular cup surface [14]. They defined the acetabular clock face using the acetabular cup geometric normal line and the transverse ligament midpoint at the inferior acetabular notch. In our study, ASA was measured along the plane perpendicular to the APP because the line of the acetabular cup surface changed after acetabular osteotomy. Therefore, the ASA was the angle relative to the pelvis, not the acetabular cup.

A limitation of this 3D evaluation method is that the positional relationship between the femoral head and pelvis is ascertained in the supine position during CT. Originally, to evaluate coverage during loading in the standing position, it was necessary to make the pelvic tilt the same as that in the standing position using a registration method that utilizes simple radiographs of the standing position, requiring two-dimensional-3D matching, i.e., standing radiographs and CT images. However, we believe that the measurement method itself is inexpensive and simple and that it can be applied to the 3D evaluation of other osteotomies in the future, such as varus osteotomy of the femur or opening wedge osteotomy around the knee.

In conclusion, by combining the new method with reference fiducials, we found that femoral head coverage, especially on the lateral side, could be achieved as planned, with fewer errors. The clinical outcomes did not differ significantly between the previous and fiducial point methods, which may be due to the short follow-up period. The effect of the improved accuracy of the fiducial point method on clinical outcomes will be investigated in the future.
